# Comprehensive analysis of the long noncoding RNA HOXA11-AS gene interaction regulatory network in NSCLC cells

**DOI:** 10.1186/s12935-016-0366-6

**Published:** 2016-12-01

**Authors:** Yu Zhang, Rong-quan He, Yi-wu Dang, Xiu-ling Zhang, Xiao Wang, Su-ning Huang, Wen-ting Huang, Meng-tong Jiang, Xiao-ning Gan, You Xie, Ping Li, Dian-zhong Luo, Gang Chen, Ting-qing Gan

**Affiliations:** 1 Department of Pathology, First Affiliated Hospital of Guangxi Medical University, No. 6 Shuangyong Road, Guangxi Zhuang Autonomous Region, Nanning, 530021 People’s Republic of China; 2 Department of Medical Oncology, First Affiliated Hospital of Guangxi Medical University, No. 6 Shuangyong Road, Guangxi Zhuang Autonomous Region, Nanning, 530021 People’s Republic of China; 3 Department of Orthopedics, China-Japan Union Hospital of Jilin University, 2 Sendai Street, Changchun, 130033 People’s Republic of China; 4 Department of Radiotherapy, First Affiliated Hospital of Guangxi Medical University, No. 6 Shuangyong Road, Guangxi Zhuang Autonomous Region, Nanning, 530021 People’s Republic of China

**Keywords:** HOXA11-AS, NSCLC, Microarray assay, GO, KEGG, Pathway

## Abstract

**Background:**

Long noncoding RNAs (lncRNAs) are related to different biological processes in non-small cell lung cancer (NSCLC). However, the possible molecular mechanisms underlying the effects of the long noncoding RNA HOXA11-AS (HOXA11 antisense RNA) in NSCLC are unknown.

**Methods:**

HOXA11-AS was knocked down in the NSCLC A549 cell line and a high throughput microarray assay was applied to detect changes in the gene profiles of the A549 cells. Bioinformatics analyses (gene ontology (GO), pathway, Kyoto Encyclopedia of Genes and Genomes (KEGG), and network analyses) were performed to investigate the potential pathways and networks of the differentially expressed genes. The molecular signatures database (MSigDB) was used to display the expression profiles of these differentially expressed genes. Furthermore, the relationships between the HOXA11-AS, de-regulated genes and clinical NSCLC parameters were verified by using NSCLC patient information from The Cancer Genome Atlas (TCGA) database. In addition, the relationship between HOXA11-AS expression and clinical diagnostic value was analyzed by receiver operating characteristic (ROC) curve.

**Results:**

Among the differentially expressed genes, 277 and 80 genes were upregulated and downregulated in NSCLC, respectively (fold change ≥2.0, P < 0.05 and false discovery rate (FDR) < 0.05). According to the degree of the fold change, six upregulated and three downregulated genes were selected for further investigation. Only four genes (RSPO3, ADAMTS8, DMBT1, and DOCK8) were reported to be related with the development or progression of NSCLC based on a PubMed search. Among all possible pathways, three pathways (the PI3K-Akt, TGF-beta and Hippo signaling pathways) were the most likely to be involved in NSCLC development and progression. Furthermore, we found that HOXA11-AS was highly expressed in both lung adenocarcinoma and squamous cell carcinoma based on TCGA database. The ROC curve showed that the area under curve (AUC) of HOXA11-AS was 0.727 (95% CI 0.663–0.790) for lung adenocarcinoma and 0.933 (95% CI 0.906–0.960) for squamous cell carcinoma patients. Additionally, the original data from TCGA verified that ADAMTS8, DMBT1 and DOCK8 were downregulated in both lung adenocarcinoma and squamous cell carcinoma, whereas RSPO3 expression was upregulated in lung adenocarcinoma and downregulated in lung squamous cell carcinoma. For the other five genes (STMN2, SPINK6, TUSC3, LOC100128054, and C8orf22), we found that STMN2, TUSC3 and C8orf22 were upregulated in squamous cell carcinoma and that STMN2 and USC3 were upregulated in lung adenocarcinoma. Furthermore, we compared the correlation between HOXA11-AS and de-regulated genes in NSCLC based on TCGA. The results showed that the HOXA11-AS expression was negatively correlated with DOCK8 in squamous cell carcinoma (r = −0.124, P = 0.048) and lung adenocarcinoma (r = −0.176, P = 0.005). In addition, RSPO3, ADAMTS8 and DOCK8 were related to overall survival and disease-free survival (all P < 0.05) of lung adenocarcinoma patients in TCGA.

**Conclusions:**

Our results showed that the gene profiles were significantly changed after HOXA11-AS knock-down in NSCLC cells. We speculated that HOXA11-AS may play an important role in NSCLC development and progression by regulating the expression of various pathways and genes, especially DOCK8 and TGF-beta pathway. However, the exact mechanism should be verified by functional experiments.

## Background

Lung cancer is the most common cancer worldwide and the first leading cause of cancer death [1, 2]. More than 1.8 million lung cancer patients are diagnosed each year, accounting for approximately 13% of newly diagnosed cancer cases [3]. Lung cancer can be divided into two categories based on the histological type [small cell lung cancer (SCLC) and non-small cell lung cancer (NSCLC)]. NSCLC accounts for 80–85% of new lung cancers. NSCLC can be divided into different subgroups, such as adenocarcinoma, squamous cell carcinoma, adenosquamous carcinoma, undifferentiated carcinoma and large cell carcinoma. More than 70% of NSCLC cases are advanced disease and the 5-year survival rate for NSCLC is only 16% [4]. Hence, research into the etiology and mechanism is of great significance for the diagnosis and treatment of lung cancer.

Long non-coding RNAs (lncRNAs) represent RNAs more than 200 nucleotides in length that lack a protein-coding capacity. Many lncRNAs have been reported to be associated with transcriptional regulation, disease development or epigenetic gene regulation [5–7]. Additionally, lncRNAs are involved in numerous biological functions, such as tumorigenesis, immune responses, cell differentiation and other biological processes [8–11]. To date, many lncRNAs have been reported to play important roles in NSCLC, such as lncRNA-TATDN1, PVT1 and MALAT1, which may influence the NSCLC cell proliferation, invasion and metastasis, respectively [12–14]. However, the biological and molecular mechanisms underlying the actions of HOXA11-AS in NSCLC have not been fully explored.

**Fig. 1:**
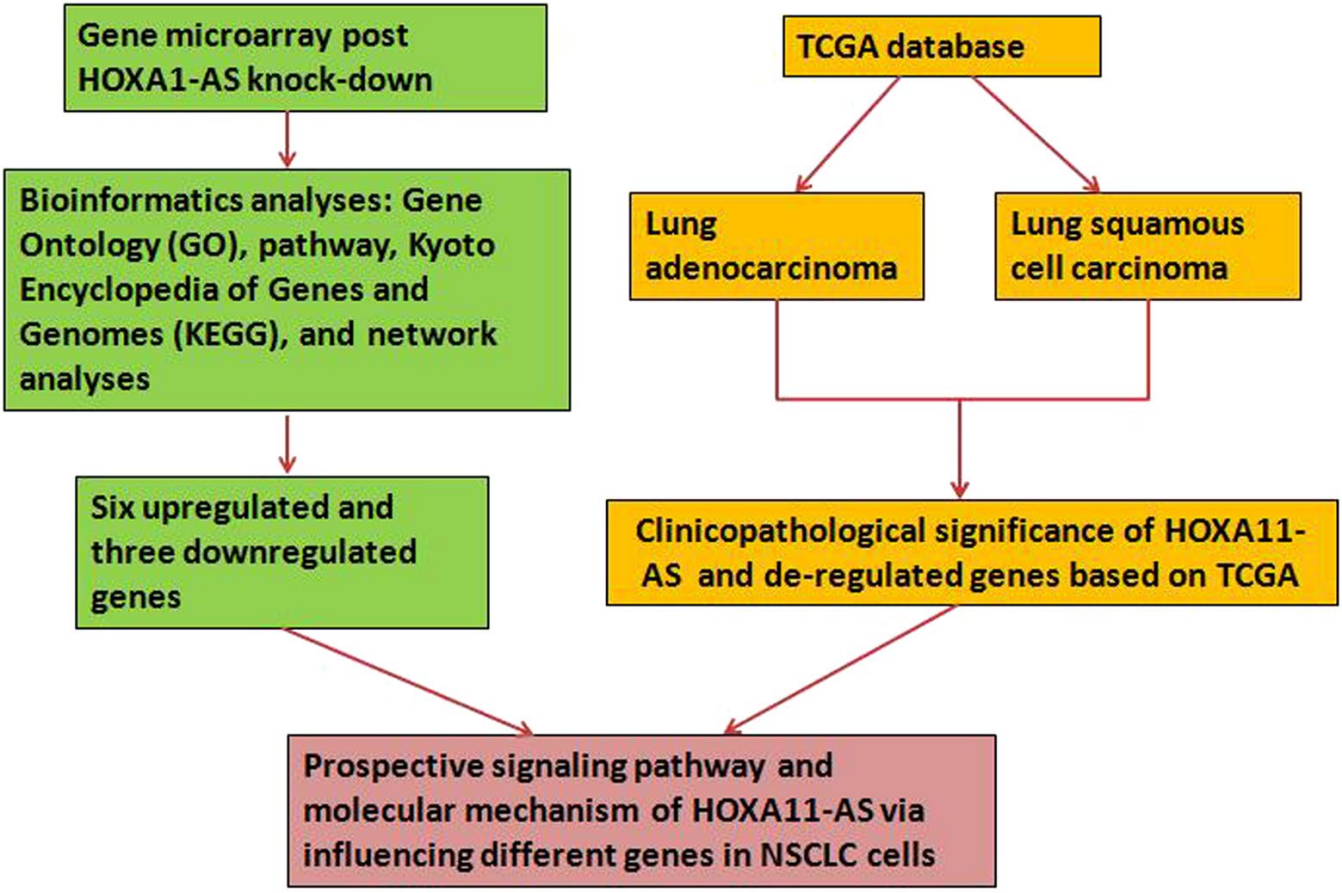
A flow chart of this study was shown

HOXA11-AS (also known as HOXA11S, HOXA-AS5, HOXA11AS, HOXA11-AS1, and NCRNA00076) is located on 7p15.2 (NCBI Gene ID: 221883). HOXA11-AS is a member of the homeobox (HOX) family of genes with a length of 3885 nt. To date, only 2 studies have reported a relationship between HOXA11-AS and cancer. Richards et al. [15] demonstrated that HOXA11-AS inhibited the oncogenic phenotype of epithelial ovarian cancer by analyzing genome-wide association study data and performing a series of functional experiments. Wang et al. [16] confirmed that HOXA11-AS was a cell cycle-associated lncRNA and could serve as a biomarker of glioma progression using a high-throughput microarray and gene set enrichment analysis. However, the expression and function of HOXA11-AS in NSCLC tissues is unknown. We designed this study to explore expression profile changes after HOXA11-AS knock-down and the possible molecular mechanisms of HOXA11-AS in NSCLC development and progression. A flow chart of this study was shown in Fig. 1.

## Methods

### Knock-down of HOXA11-AS in the NSCLC cell A549 and transfection with HOXA11-AS-siRNA

The human NSCLC A549 cell line was purchased from the Type Culture Collection of the Chinese Academy of Sciences (Shanghai, China). NSCLC A549 cells were cultivated with 10% heat-inactivated fetal bovine serum (Invitrogen Corp, Grand Island, NY, USA) in a humidified 5% CO_2_ atmosphere with 2 mM glutamine and gentamicin at 37 °C. Three DcR3-specific siRNAs were synthesized by GenePharma (Shanghai, China) and merged into one siRNA pool (Table 1). The NSCLC A549 cell line was transfected with the HOXA11-AS-siRNA. The CombiMAG magnetofection reagent (OZ BIOSCIENCES, Marseille, France) was used for the transfection according to the manufacturer’s instructions.

**Table 1:** The HOXA11-AS-siRNA sequences

ID	Target sequence	GC %
HOXA11-AS-RNAi(32154-2)	CTACCATCCCTGAGCCTTA	52.63
HOXA11-AS-RNAi(32155-1)	TGACATCCGAGGAGACTTC	52.63
HOXA11-AS-RNAi(32156-1)	CGTAATCGCCGGTGTAACT	52.63

### Microarray analysis and computational analysis

**Fig. 2:**
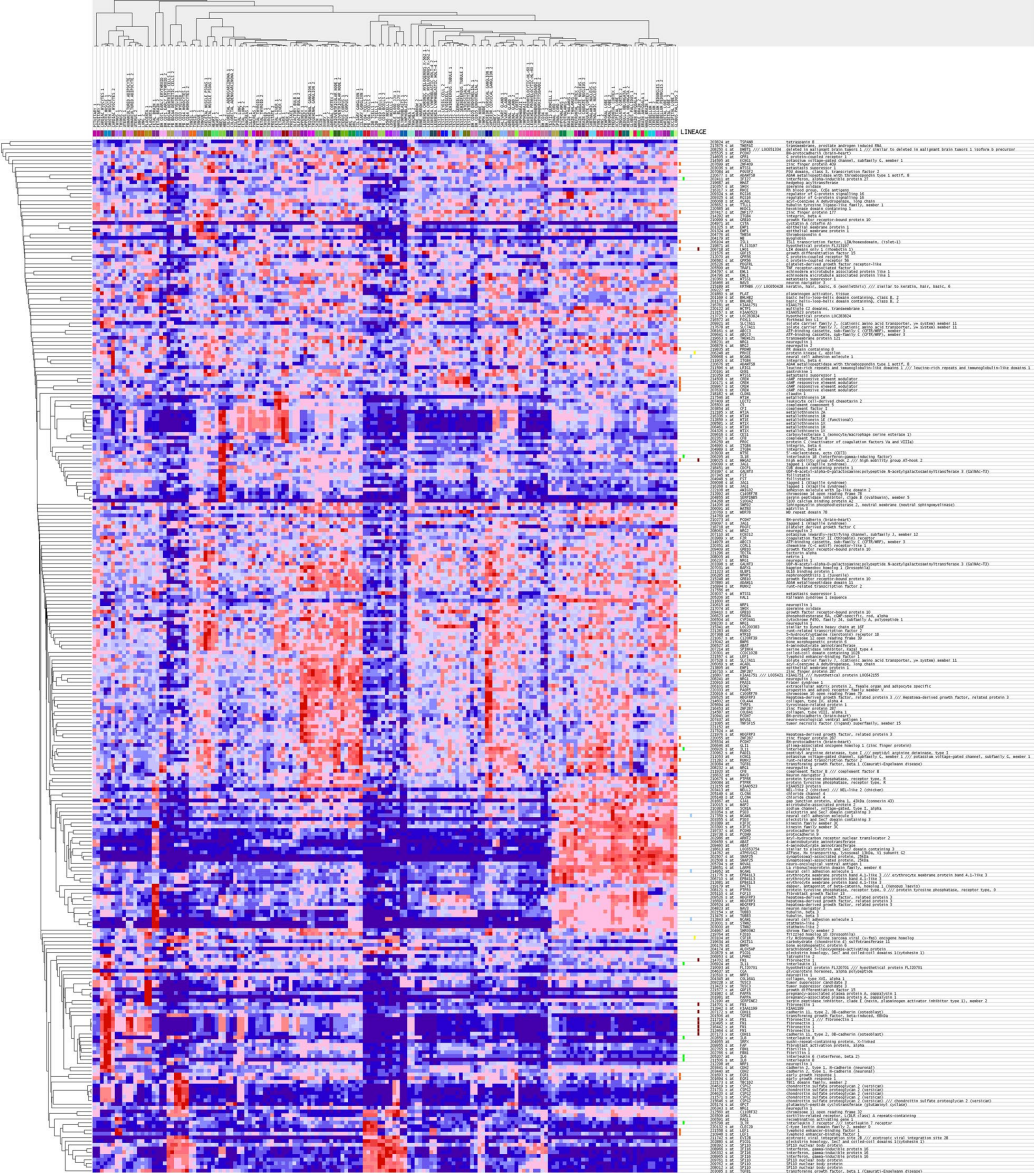
Hierarchical clustering (*heat map*) of transcript expression for the 280 upregulated genes with the most differential expression between tumors

**Fig. 3:**
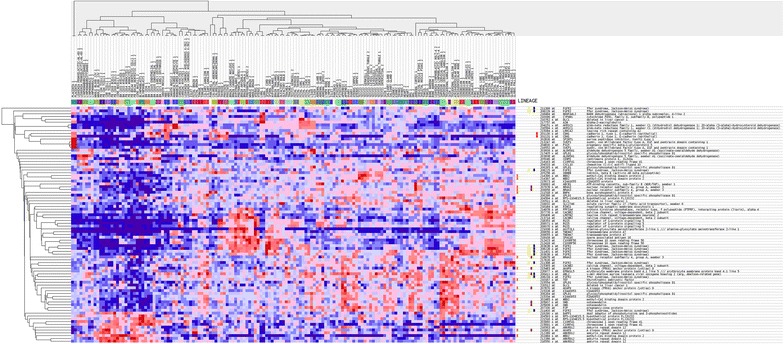
Hierarchical clustering (*heat map*) of transcript expression for the 80 downregulated genes with the most differential expression between tumors

The sample analysis and microarray hybridization were performed by Kangchen Bio-tech (Shanghai, China). Briefly, RNA was purified and extracted from 1 mg of total RNA after removing the rRNA (mRNA-ONLY Eukaryotic mRNA Isolation Kit, Epicentre Biotechnologies, Madison, USA). Then, each sample was transcribed and amplified into fluorescent cRNA using a random priming method. The cRNAs were labeled and hybridized onto the Human MRNA Array v3.0 (8 × 60 K, Arraystar, Rockville, MD, USA). After washing the slides, the arrays were scanned with the Agilent Scanner G2505C. The Agilent Feature Extraction software (version 11.0.1.1) was used to analyze the acquired array images. Quantile normalization and subsequent data processing were implemented by the GeneSpring GX v11.5.1 software package (Agilent Technologies). Differentially expressed genes were identified based on fold change filtering (fold change ≥2.0 or ≤0.5), a paired *t* test (p < 0.05) and multiple hypothesis testing (FDR < 0.05). The P values and FDR were calculated with Microsoft Excel and MATLAB, respectively. Differentially expressed genes between the RNAi and control samples were identified with an absolute fold change >2 as the cut-off. The molecular signatures database (MSigDB, http://www.broadinstitute.org/msigdb) was applied to visualize the expression profiles of these differentially expressed genes (Figs. 2, 3).

### GO analysis and pathway analysis

To better understand the potential roles of the differentially expressed genes, gene ontology (GO) analysis and pathway analysis were performed as previously described [17]. In this process, we included the following three independent categories derived from the GO Consortium website (http://www.geneontology.org): biological process (BP), cellular component (CC) and molecular function (MF) [17]. The enrichment of the upregulated and downregulated coding genes was analyzed by uploading the datasets to the database for annotation, visualization and integrated discovery (DAVID, http://david.abcc.ncifcrf.gov/). The Kyoto Encyclopedia of Genes and Genomes (KEGG) database (http://www.genome.jp/kegg/) was used to analyze the biological pathways where there was an obvious enrichment of differentially expressed genes [18].

### Additional analysis of 9 de-regulated genes in NSCLC from TCGA

TCGA is a collection of exome sequencing, DNA methylation, SNP array, miRNA-seq, and RNA-seq data [19]. TCGA can be used to analyze complicated clinical profiles and cancer genomics [20, 21]. In this study, original expression data for HOXA11-AS and the 9 genes de-regulated in lung adenocarcinoma and squamous cell carcinoma were extracted from TCGA and analyzed. Additionally, original data for cancerous or non-cancerous lung tissues were downloaded and analyzed. Also, the relationship between HOXA11-AS expression and clinical diagnostic value was analyzed by receiver operating characteristic (ROC) curve. Besides, we extracted the co-genes of HOXA11-AS from TCGA through R Project for Statistical Computing (https://www.r-project.org/). Genes with a FDR < 0.05 was considered for co-expressed relationship.

### Statistical analysis

SPSS 20.0 was applied for the statistical analysis. The Mann–Whitney U test was used to compare the expression of the four de-regulated genes in terms of different clinical features (age, gender, TNM stage, tumor size, distant metastasis and lymph node metastasis). P < 0.05 was considered statistically significant (two-sides).

## Results

### Gene expression profiles regulated by the HOXA11-AS lncRNA

**Fig. 4:**
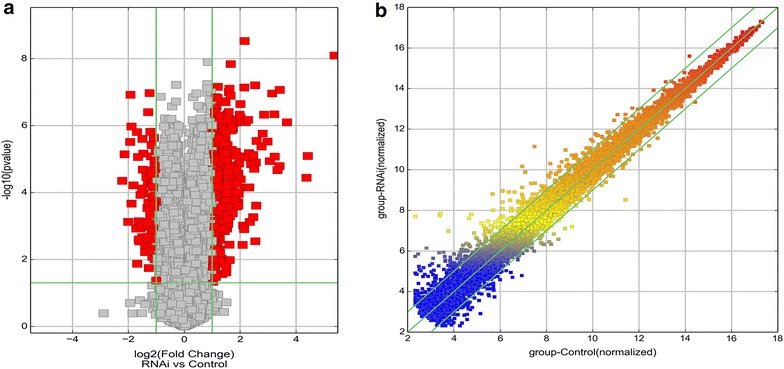
Gene clip after HOXA11-AS knock-down in NSCLC. **a** *Volcano plot*; **b** *box*-*scatter plot*

A high throughput microarray assay was applied to detect differential expression profiles between HOXA11-AS and HOXA11-AS RNAi in three paired A549 cell cultures. Thirteen thousand three hundred and twenty-three upregulated genes and 14,384 downregulated genes were differentially expressed in the HOXA11-AS-control and HOXA11-AS-RNAi groups. A summary of these differentially expressed genes is presented in Fig. 4. The fold changes (HOXA11-AS-control vs HOXA11-AS-RNAi) and P values were calculated using the normalized expression values. Using microarray analysis, 357 genes were identified as significantly differentially expressed in NSCLC compared with the RNAi control samples (fold change ≥ 2.0, P < 0.05 and FDR < 0.05). Among them, 277 genes were upregulated in all three NSCLC groups and 80 genes were downregulated. Furthermore, the number of aberrantly expressed genes varied with the different fold changes (Table 2). Among them, 16 genes were upregulated by more than sixfold in the HOXA11-AS compared to the HOXA11-AS RNAi samples and 3 genes were downregulated by more than fourfold. 6 of the 15 upregulated genes were upregulated by more than tenfold (Table 2). The top 6 upregulated and top 3 downregulated genes are shown in Table 3. Among these 9 aberrantly expressed genes, the expression of RSPO3 (NM_032784, fold change = 41.610487, P = 8.0502E−09) was dramatically upregulated and the expression of LOC100128054 (NR_033969, fold change = 4.6652225, P = 4.45517E−05) was significantly downregulated. When we searched PubMed (http://www.ncbi.nlm.nih.gov/pubmed) to identify reported functions for these differentially expressed genes, we found that only 4 genes (RSPO3, ADAMTS8, DMBT1, and DOCK8) were reported to be associated with NSCLC. RSPO3 was reported to promote tumor aggressiveness in Keap1-deficient lung adenocarcinomas [22]. ADAMTS8 was related to promoter hyper methylation in early-stage NSCLCs [23, 24]. DMBT1 was a candidate tumor suppressor gene; DMBT1 expression is often lost in lung cancer, indicating that DMBT1 inactivation may have a significant influence on lung tumorigenesis [25]. DOCK8 was suggested to be involved in the development and/or progression of lung cancer [26]. Thus, these genes may play essential roles in the occurrence and development of NSCLC.

**Table 2:** Number of aberrantly expressed genes in the microarray

	Fold change 2–4	Fold change 4–6	Fold change > 6	Total
Upregulated	238	23	16	277
Downregulated	77	3		80

**Table 3:** The top 6 upregulated and top 3 downregulated genes

Genbank accession	Gene symbol	Fold change	P
Upregulated genes			
NM_032784	RSPO3	41.610487	8.05E−09
NM_007037	ADAMTS8	21.38143	8.074E−06
NM_007029	STMN2	20.713397	3.617E−05
NM_007329	DMBT1	12.747211	7.941E−07
NM_205841	SPINK6	10.662449	8.553E−08
NM_178234	TUSC3	10.584962	1.619E−05
Downregulated genes			
NR_033969	LOC100128054	4.6652225	4.455E−05
NM_203447	DOCK8	4.4005987	7.142E−06
NM_001007176	C8orf22	4.0156588	0.000753

### GO and pathway analysis

**Fig. 5:**
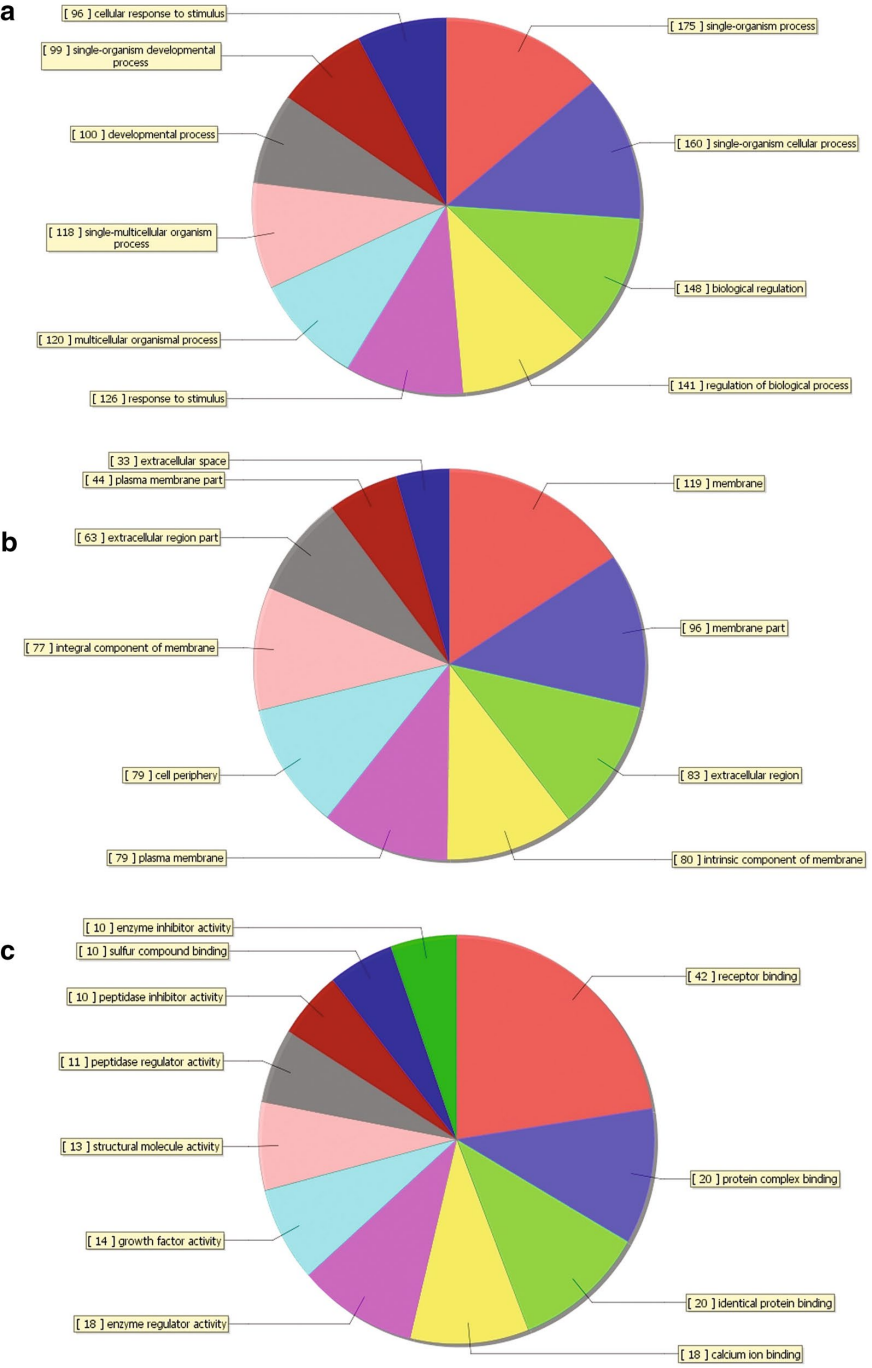
Distribution of gene ontology (GO) terms for the upregulated genes in NSCLC. The *pie plot* showing the gene ontology classification for the upregulated genes in NSCLC. The graph does not contain all upregulated genes because the majority do not have assigned GOs. **a** Biological process (BP). **b** Cellular component (CC). **c** Molecular function (MF)

**Fig. 6:**
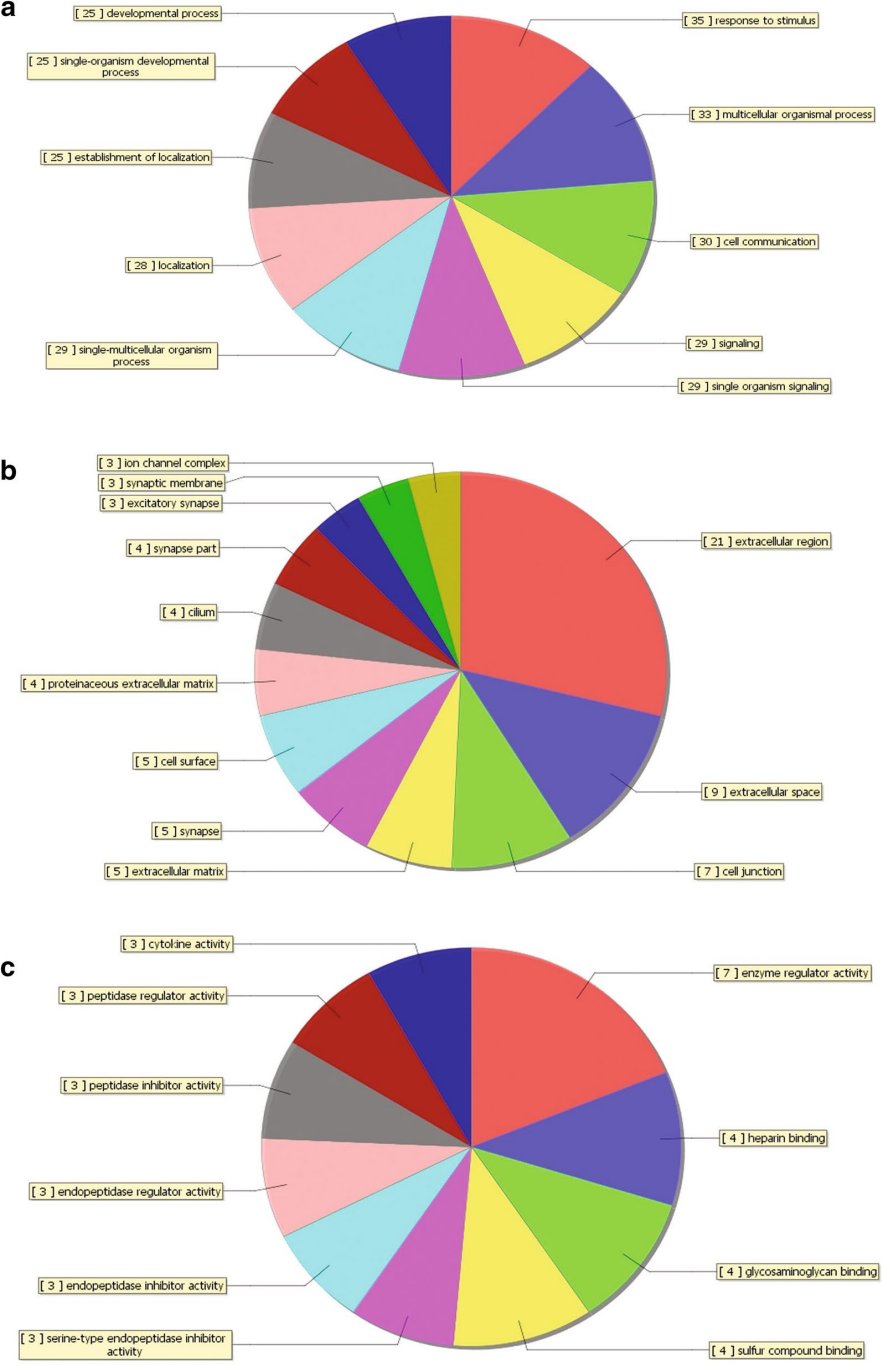
Distribution of gene ontology (GO) terms for the downregulated genes in NSCLC. The *pie plot* showing the gene ontology classification for the downregulated genes in NSCLC. The graph does not contain all downregulated genes because the majority do not have assigned GOs. **a** Biological process (BP). **b** Cellular component (CC). **c** Molecular function (MF)

**Fig. 7:**
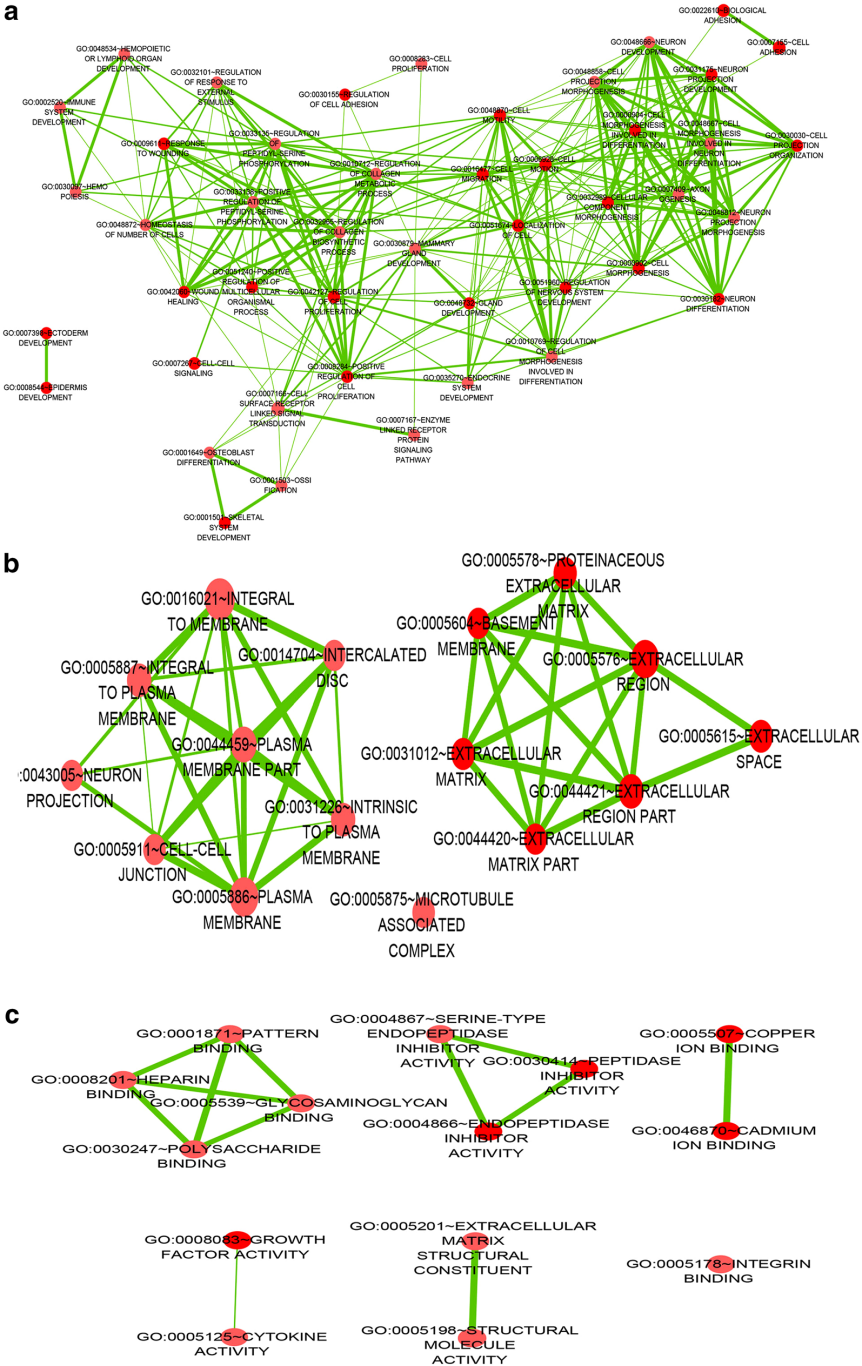
A function network of gene ontology (GO) terms for the upregulated genes in NSCLC. **a** Biological process (BP). **b** Cellular component (CC). **c** Molecular function (MF)

**Fig. 8:**
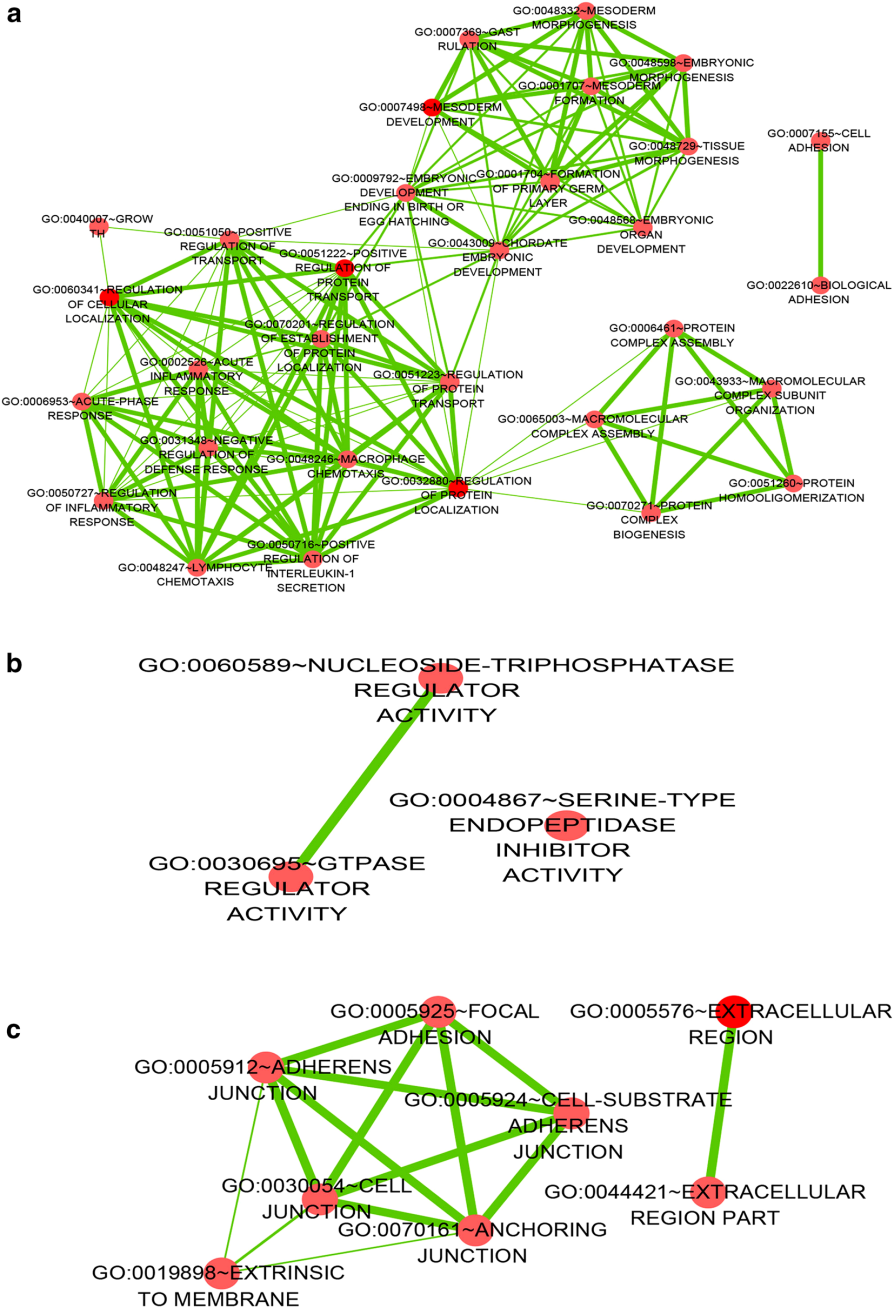
A function network (BP) of Gene Ontology (GO) terms for the downregulated genes in NSCLC. *BP* biological process

The GO analysis identified biological processes, molecular functions and cellular components in which the differentially expressed genes may be involved. The top five most enriched GO terms are shown in Table 4. The GO analysis results clarified the most significant functional groups, such as single-organism process, cellular response to stimulus, biological regulation, and cellular component organization (Figs. 5, 6). To better understand the relevant functions of these genes, a function network was constructed based on the GO analysis (Figs. 7, 8). We constructed only the BP ontology for the downregulated genes because only 80 genes were identified (Fig. 8).

**Table 4:** Top 5 enriched GO terms (BP, CC, and MF) from the microarray data

GO.ID	Term	Ontology	Enrichment score	P
Upregulated genes				
GO:0071294	Cellular response to zinc ion	BP	8.557911479	2.76751E−09
GO:0044707	Single-multicellular organism process	BP	8.514757717	3.05663E−09
GO:0048731	System development	BP	8.400063137	3.98049E−09
GO:0032501	Multicellular organismal process	BP	8.203661346	6.2566E−09
GO:0048856	Anatomical structure development	BP	8.143712557	7.1827E−09
GO:0005578	Proteinaceous extracellular matrix	CC	8.300801864	5.00263E−09
GO:0031012	Extracellular matrix	CC	8.221562341	6.00396E−09
GO:0005604	Basement membrane	CC	6.791104942	1.61769E−07
GO:0044420	Extracellular matrix part	CC	5.570158827	2.69055E−06
GO:0005576	Extracellular region	CC	5.516943306	3.04128E−06
GO:0005102	Receptor binding	MF	8.517557576	3.03698E−09
GO:0008083	Growth factor activity	MF	7.9661485	1.08106E−08
GO:0005178	Integrin binding	MF	4.513665828	3.06432E−05
GO:0030414	Peptidase inhibitor activity	MF	4.359313435	4.37206E−05
GO:0061134	Peptidase regulator activity	MF	4.350560953	4.46107E−05
Downregulated genes				
GO:0001707	Mesoderm formation	BP	4.07566169	8.40114E−05
GO:0048332	Mesoderm morphogenesis	BP	4.002278949	9.94766E−05
GO:0016331	Morphogenesis of embryonic epithelium	BP	3.806552741	0.000156116
GO:0048729	Tissue morphogenesis	BP	3.403951508	0.000394501
GO:0002064	Epithelial cell development	BP	3.374049298	0.000422621
GO:0060076	Excitatory synapse	CC	4.66716144	2.15198E−05
GO:0034364	High-density lipoprotein particle	CC	2.577293133	0.002646713
GO:0048786	Presynaptic active zone	CC	2.543382451	0.002861657
GO:0034358	Plasma lipoprotein particle	CC	2.241145559	0.005739241
GO:0032994	Protein-lipid complex	CC	2.196499993	0.006360628
GO:0008201	Heparin binding	MF	3.006284448	0.000985634
GO:0005539	Glycosaminoglycan binding	MF	2.534599082	0.002920121
GO:0004867	Serine-type endopeptidase inhibitor activity	MF	2.466282926	0.003417567
GO:1901681	Sulfur compound binding	MF	2.39941613	0.003986427
GO:0004866	Endopeptidase inhibitor activity	MF	1.829660366	0.014802656

The KEGG analysis showed that the aberrantly expressed genes might be related to different pathways. A total of 21 upregulated pathways and only 1 downregulated pathway were available through the pathway analysis. The most important enriched pathway terms are shown in Table 5 (P_upregulated_ < 0.01). Three pathways (PI3K-Akt signaling pathway, TGF-beta signaling pathway and Hippo signaling pathway) were previously reported to be involved in NSCLC development and progression. As reported, the PI3K-Akt signaling pathway was related to NSCLC cell proliferation, apoptosis and autophagy [27–29]. The TGF-beta signaling pathway could be associated with the NSCLC cell DNA damage response, radiation sensitivity, viability and invasion capacity [30, 31]. The Hippo signaling pathway was involved in NSCLC cell migration and invasion [32].

**Table 5:** The most important enriched pathway terms from the microarray data

Pathway ID	Definition	Enrichment_Score	P
Upregulated genes			
hsa04978	Mineral absorption—*Homo sapiens* (human)	4.539769	2.88557E−05
hsa04610	Complement and coagulation cascades—*Homo sapiens* (human)	3.791003	0.000161807
hsa05200	Pathways in cancer—*Homo sapiens* (human)	3.329344	0.000468443
hsa05020	Prion diseases—*Homo sapiens* (human)	3.073759	0.000843803
hsa05144	Malaria—*Homo sapiens* (human)	2.569023	0.002697597
hsa05217	Basal cell carcinoma—*Homo sapiens* (human)	2.385839	0.00411302
hsa05323	Rheumatoid arthritis—*Homo sapiens* (human)	2.34211	0.004548726
hsa04060	Cytokine-cytokine receptor interaction—*Homo sapiens* (human)	2.310768	0.004889133
hsa05202	Transcriptional misregulation in cancer—*Homo sapiens* (human)	2.264309	0.005441153
hsa04151	PI3K-Akt signaling pathway—*Homo sapiens* (human)	2.098056	0.007978919
Downregulated genes			
hsa04350	TGF-beta signaling pathway—*Homo sapiens* (human)	1.561561	0.02744347

P_upregulated_ < 0.01

**Fig. 9:**
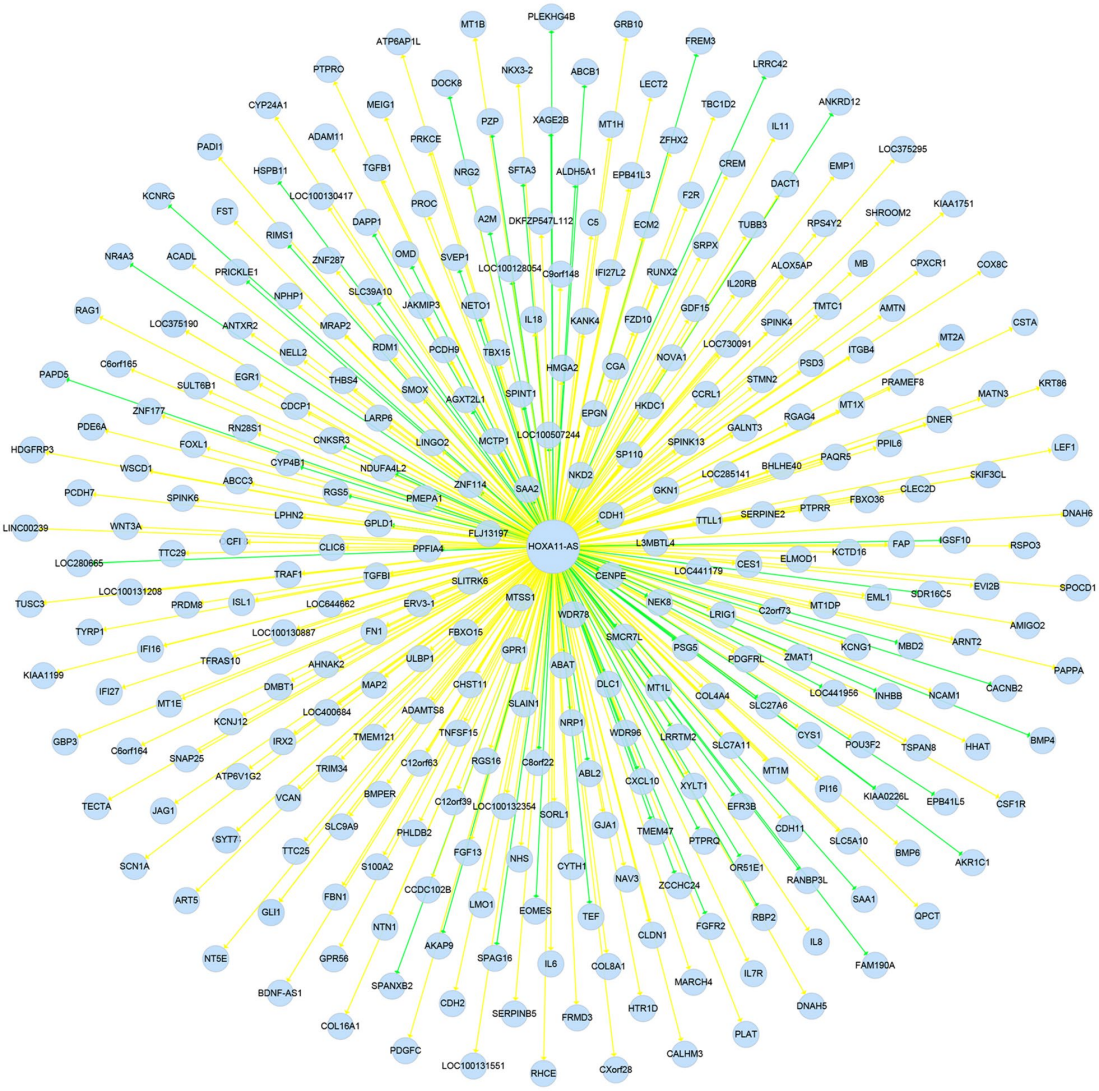
Network analysis between HOXA11-AS and the differentially expressed genes. *Yellow* indicates activation and *green* indicates inhibition

A gene network of these 357 genes was constructed in the present study (Fig. 9). The relationships between HOXA11-AS and the differentially expressed genes were easily observed from this network analysis.

### Supplementary information from the TCGA

**Fig. 10:**
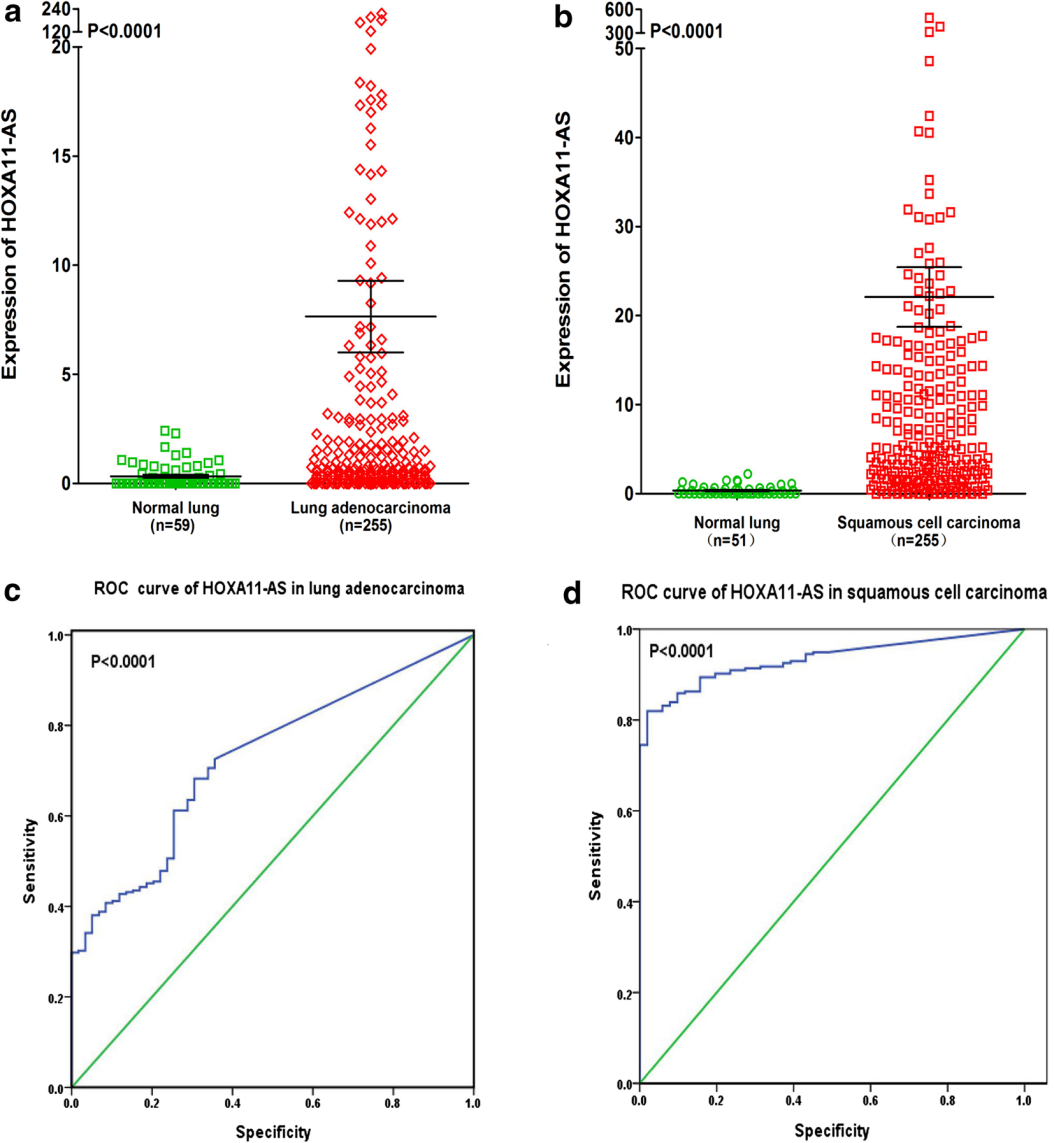
Differential expression and ROC curve of HOXA11-AS in lung adenocarcinoma and squamous cell carcinoma based on The Cancer Genome Atlas (TCGA) database. **a** Differential expression of HOXA11-AS in lung adenocarcinoma. **b** Differential expression of HOXA11-AS in squamous cell carcinoma. **c** ROC curve of HOXA11-AS in lung adenocarcinoma. **d** ROC curve of HOXA11-AS in squamous cell carcinoma

In order to explore the relationship between HOXA11-AS expression and NSCLC, we performed a clinical study with the original data in TCGA. We found that HOXA11-AS was upregulated in both lung adenocarcinoma and squamous cell carcinoma compared to non-cancerous lung tissues (both P < 0.0001, Fig. 10a, b). And the ROC curve revealed that the area under curve (AUC) of HOXA11-AS was 0.727 (95% CI 0.663–0.790) for lung adenocarcinoma patients and 0.933 (95% CI 0.906–0.960) for squamous cell carcinoma patients (both P < 0.0001), which could gain high diagnostic value of HOXA11-AS level in NSCLC (Fig. 10c, d).

**Fig. 11:**
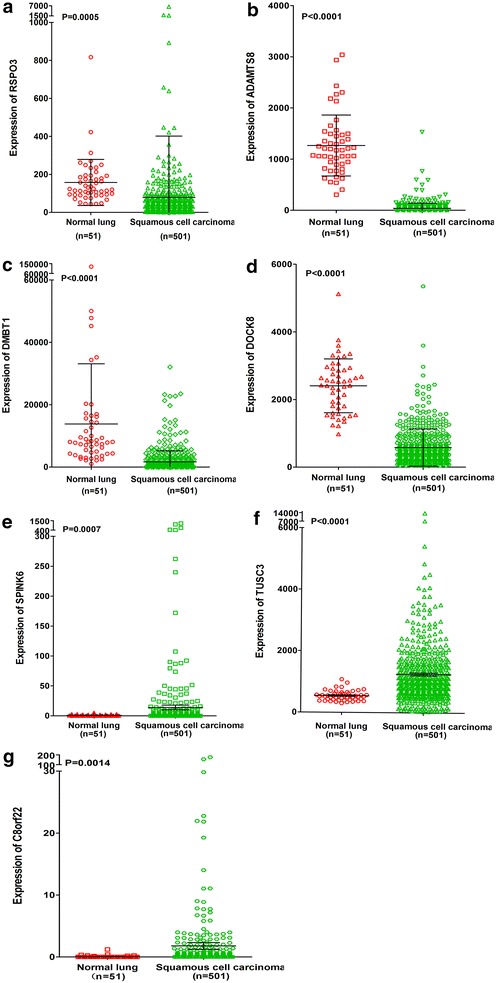
Differential expression of genes between squamous cell carcinoma and normal lung tissues based on The Cancer Genome Atlas (TCGA) database. **a** RSPO3; **b** ADAMTS8; **c** DMBT1; **d** DOCK8; **e** SPINK6; **f** TUSC3; **g** C8orf22

**Fig. 12:**
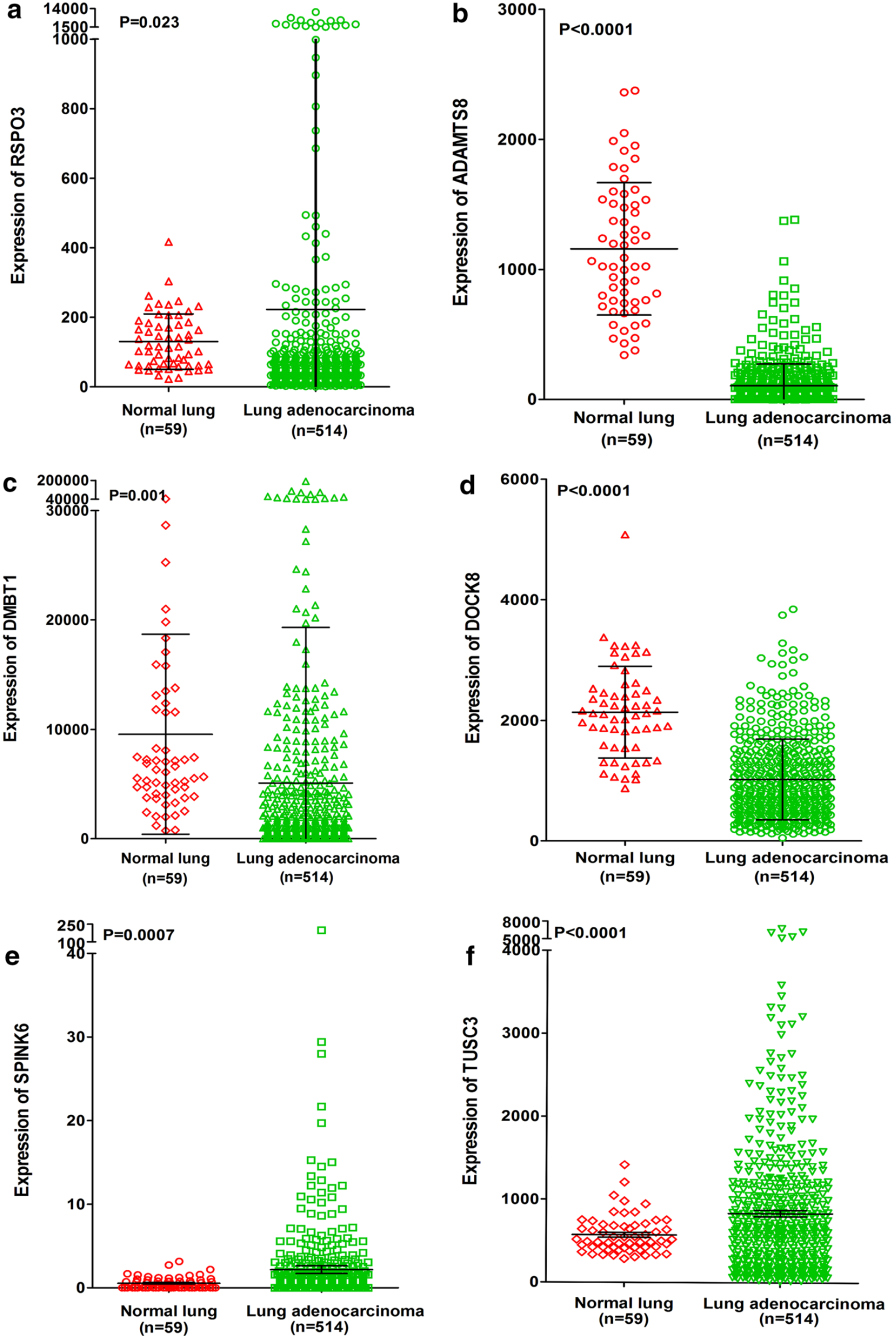
Differential expression of RSPO3, ADAMTS8, DMBT1 and DOCK8 between lung adenocarcinoma and normal lung tissues based on The Cancer Genome Atlas (TCGA) database. **a** RSPO3; **b** ADAMTS8; **c** DMBT1; **d** DOCK8; **e** SPINK6; **f** TUSC3

To elucidate the relationships between the 9 de-regulated genes and NSCLC, we searched the original data from 514 adenocarcinoma cases and 501 squamous cell carcinoma cases in TCGA. We also compared gene expression between adenocarcinoma, squamous cell carcinoma and non-cancerous lung tissues. We found that RSPO3, ADAMTS8, DMBT1 and DOCK8 were all downregulated in squamous cell carcinoma tissues compared to non-cancerous lung tissues, whereas STMN2, TUSC3 and C8orf22 were upregulated in squamous cell carcinoma (all P < 0.001, Fig. 11). Additionally, ADAMTS8, DMBT1 and DOCK8 were down-regulated in adenocarcinoma and STMN2 and TUSC3 were up-regulated in lung adenocarcinoma (all P < 0.01, Fig. 12). RSPO3 was overexpressed in adenocarcinoma but not squamous cell carcinoma (P = 0.023).

Furthermore, we compared the correlation between HOXA11-AS and de-regulated genes in NSCLC based on TCGA. The results showed that the HOXA11-AS expression was negatively correlated with DOCK8 in squamous cell carcinoma (r = −0.124, P = 0.048) and lung adenocarcinoma (r = −0.176, P = 0.005). No obviously correlation was found between HOXA11-AS and other de-regulated genes (Table 6). Besides, the co-genes of HOXA11-AS in TCGA was extracted through R Project for Statistical Computing. We found that RSPO3, ADAMTS8, DMBT1, DOCK8, STMN2, SPINK6 and TUSC3 were the co-genes of HOXA11-AS in lung adenocarcinoma whereas RSPO3, ADAMTS8, DMBT1, DOCK8, STMN2, SPINK6, TUSC3 and C8orf22 were the co-genes of HOXA11-AS in squamous cell carcinoma.

**Table 6:** The correlation between HOXA11-AS and de-regulated genes in NSCLC based on TCGA

Gene symbol	Squamous cell carcinoma		Lung adenocarcinoma	
	R	P	R	P
RSPO3	−0.022	0.724	−0.025	0.656
ADAMTS8	−0.107	0.087	−0.020	0.756
STMN2	0.000	0.996	0.049	0.434
DMBT1	−0.061	0.330	−0.064	0.309
SPINK6	−0.007	0.911	−0.034	0.589
TUSC3	−0.014	0.828	0.013	0.839
DOCK8	−0.176	0.005	−0.124	0.048
C8orf22	−0.007	0.910	−0.025	0.656

**Fig. 13:**
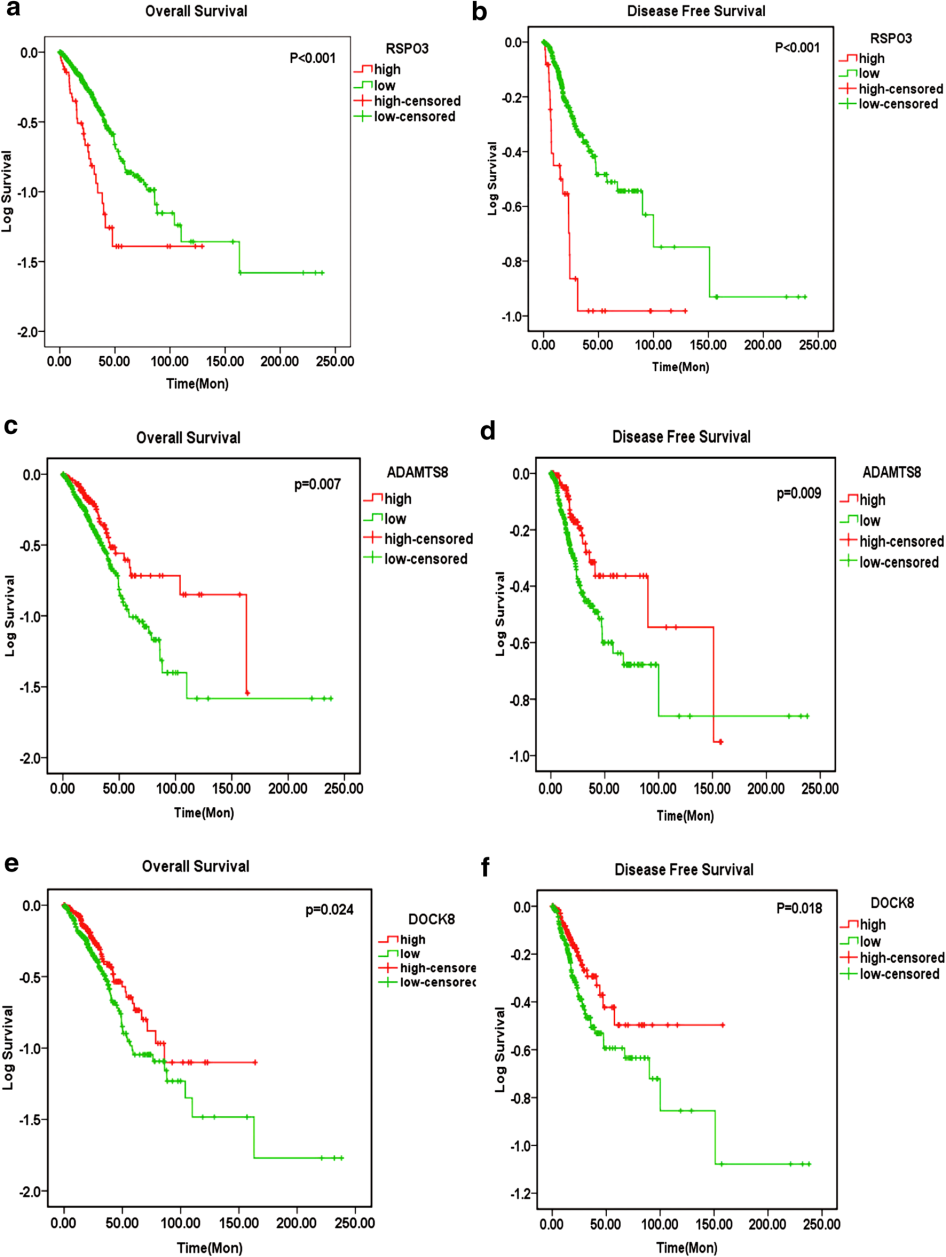
Kaplan-Meyer curves of RSPO3, ADAMTS8 and DOCK8 expression in lung adenocarcinoma based on The Cancer Genome Atlas (TCGA) database. **a** Overall survival of RSPO3 in lung adenocarcinoma. Patients with high RSPO3 expression had a significantly poorer prognosis (46.749 ± 7.528 months) than those with low expression (90.101 ± 8.759 months, P < 0.0001). **b** Disease-free survival of RSPO3 in lung adenocarcinoma. Patients with high RSPO3 expression had a significantly poorer prognosis (56.254 ± 10.462 months) than those with low expression (127.159 ± 13.180, P < 0.0001). **c** Overall survival of ADAMTS8 in lung adenocarcinoma. Patients with low ADAMTS8 expression had a significantly poorer prognosis (80.869 ± 8.989 months) than those with high expression (92.497 ± 8.863 months, P = 0.007). **d** Disease-free survival of ADAMTS8 in lung adenocarcinoma. Patients with high ADAMTS8 expression had a significantly poorer prognosis (107.704 ± 10.239 months) than those with low expression (121.080 ± 14.027 months, P = 0.009). **e** Overall survival of DOCK8 in lung adenocarcinoma. Patients with low DOCK8 expression had a significantly poorer prognosis (80.028 ± 9.108 months) than those with high expression (81.730 ± 8.029 months, P = 0.024). **f** Disease-free survival of DOCK8 in lung adenocarcinoma. Patients with high DOCK8 expression had a significantly poorer prognosis (107.246 ± 8.779 months) than those with high expression (114.254 ± 13.518 months, P = 0.024)

In addition, we also investigated the relationship between the expression levels of the de-regulated genes and clinicopathological parameters or patient survival. Only ADAMTS8 was related to the TNM stage (t = 0.041, P = 0.032) in squamous cell carcinoma. In lung adenocarcinoma tissues, RSPO3 was obviously more highly expressed in the advanced stages (III and IV) than the early stages (I–II, t = −2.462, P = 0.015). When lymph node metastasis was analyzed, higher RSPO3 expression was found in cases with lymph node metastasis than in cases without (t = −2.346, P = 0.020). We also found that higher ADAMTS8 expression was more common in females (t = −2.924, P = 0.004) and cases with distant metastasis (P = 0.045). Higher DMBT1 and DOCK8 expression was also more common in females than males (all P < 0.05). DOCK8 was significantly more highly expressed in the advanced stages (III and IV, t = 3.482, P = 0.001) and cases with lymph node metastasis (t = 2.087, P = 0.037). Additionally, TUSC3 was related to age (P = 0.037). The upregulated expression of RSPO3, ADAMTS8 and DOCK8 was associated with the overall survival (all P < 0.05) and disease-free survival of adenocarcinoma patients (all P < 0.05, Fig. 13), which indicated that RSPO3, ADAMTS8 and DOCK8 might influence the prognosis of adenocarcinoma. Based on the aforementioned results, we speculated that HOXA11-AS may play an important role in NSCLC development and progression by regulating the expression of various pathways and genes, especially DOCK8 and TGF-beta pathway. However, the exact mechanism should be verified by functional experiments.

## Discussion

Lung cancer is the most common malignancy in humans and accounts for approximately 13% of newly diagnosed cancer cases per year [1–3]. NSCLC accounts for 80–85% of all lung cancers. Over the past decades, the possible molecular mechanism underlying NSCLC has been extensively explored. However, the particular pathogenesis of NSCLC is still vague. Growing evidence indicates that lncRNAs may play important roles in regulating gene expression in NSCLCs. For example, lncRNA-TATDN1 is associated with NSCLC invasion and metastasis by influencing E-cadherin, HER2, β-catenin and Ezrin expression [12], lncRNA-PVT1 promotes NSCLC cell proliferation by epigenetically regulating LATS2 expression [13] and lncRNA-MALAT1 influences tumor invasion in NSCLC by regulating DNA methylation [14]. In this study, we explored the possible biological and molecular mechanisms of HOXA11-AS in NSCLC. A microarray assay, various bioinformatics analyses (GO, pathway, KEGG, and network analyses) and the original data in TCGA were used to study differentially expressed genes and their relationships with NSCLC. After analyzing the original data from TCGA database, we found that HOXA11-AS was upregulated in both lung adenocarcinoma and squamous cell carcinoma. Also, ROC curve showed that HOXA11-AS expression might have an important value in diagnosis of lung cancer. Moreover, we searched Oncomine (https://www.oncomine.org/resource/login.html) and gene expression omnibus (GEO; http://www.ncbi.nlm.nih.gov/geo/) database, but no positively relationship was found. In addition, through the above-mentioned bioinformatics analyses, 4 genes (RSPO3, ADAMTS8, DMBT1, and DOCK8) and 3 pathways (PI3K-Akt signaling pathway, TGF-beta signaling pathway and Hippo signaling pathway) were identified as related to NSCLC. The original data from TCGA verified that ADAMTS8, DMBT1 and DOCK8 were down-regulated in adenocarcinoma and squamous cell carcinoma, whereas RSPO3 was overexpressed in adenocarcinoma and down-regulated in squamous cell carcinoma. Furthermore, RSPO3, ADAMTS8 and DOCK8 were also related to the overall survival and disease-free survival of lung adenocarcinoma patients in the TCGA data. Besides, we found that the HOXA11-AS expression was negatively correlated with DOCK8 both in squamous cell carcinoma and lung adenocarcinoma. Therefore, we hypothesized that HOXA11-AS might play an essential role in NSCLC development and progression by regulating DOCK8 expression through TGF-beta pathway. However, the real mechanism should be verified by functional experiments.

During the process of researching the relationship between these 4 de-regulated genes and 3 pathways, we found that DOCK8 and the TGF-beta signaling pathway played significant roles in the metastasis of lung adenocarcinoma [33]. Yu et al. [33] used RNA and protein analysis, Rac1 activity, imaging, cellular assays, public data set analysis and xenograft mouse models to show that DOCK4 played an important role in mediating TGF-beta-driven lung adenocarcinoma cell extravasation and metastasis. Thus, DOCK4 may act as a key component of the TGF-beta pathway. Additionally, we found that DOCK8 and the Hippo signaling pathway could play a role in neuroblastoma relapse [34]. DOCK8 mutations and YAP activation were reported to be associated with neuroblastoma relapse in one study. YAP is a member of the Hippo signaling pathway [35]; however, whether the expression of DOCK8 plays a role in NSCLC through the Hippo signaling pathway is unknown. DOCK8 (also known as MRD2, ZIR8 and HEL-205) is located on 9p24.3 (NCBI Gene ID: 81704). DOCK family proteins have been confirmed to play roles in the regulation of cell morphology, adhesion, migration and growth [36–39]. DOCK8 was reported to expressed in different cancers, such as hepatocellular carcinoma and some epithelial cancers [40, 41]. However, to date only 2 papers have reported roles for DOCK8 in lung cancer. Kang et al. [42] analyzed 22 lung squamous cell carcinoma cases and found that the loss of chromosome 9 p was specific for lung squamous cell carcinoma; thus, the DOCK8 gene might be a potential target for therapeutic measures against lung squamous cell carcinoma. Takahashi et al. [26] found that genetic and epigenetic inactivation of DOCK8 was related to the development and/or progression of lung cancer using an array-CGH analysis. The original data from TCGA verified that higher DOCK8 expression was related to gender, TNM stage, lymph node metastasis and survival, which indicated that DOCK8 might play a significant role in NSCLC. We found that the TGF-beta signaling pathway was related to radiation sensitivity, extravasation, metastasis and apoptosis [30, 33, 43]. Additionally, the deregulation of the Hippo signaling pathway induced tumors in model organisms and occurred in different human carcinomas, including lung, ovarian, colorectal and liver cancers [44]. The Hippo signaling pathway controls organ size by regulating the cell cycle, proliferation, and apoptosis [45, 46]. However, numerous in vivo and in vitro experiments need to be performed to verify whether HOXA11-AS plays a role in NSCLC development and progression by regulating DOCK8 expression through the TGF-beta or Hippo signaling pathway.

Other differentially expressed genes and pathways were investigated. Several studies have reported the functions of these genes and pathways. Gong et al. [22] found that RSPO3 was aberrantly overexpressed in half of Keap1-deficient lung adenocarcinomas and that RSPO3 overexpression resulted in much poorer survival. In vitro experiments verified that RSPO3 overexpression was related to cell proliferation and migration. The findings of these authors suggest that RSPO3 overexpression may potentially act as a driving mechanism behind the aggressiveness of Keap1-deficient lung adenocarcinomas. Dunn et al. [24] performed a microarray analysis combined with comparative multiplex RT-PCR, immunohistochemical studies and DNA methylation analysis and found that ADAMTS8 was down-regulated in primary NSCLC. ADAMTS8 down-regulation was related to promoter hypermethylation, which might be associated with NSCLC development. Mollenhauer et al. [47] explored DMBT1 expression in normal and lung cancer tissues using reverse-transcription PCR and immunohistochemical studies and found DMBT1 down-regulation in the lung cancer cell lines. However, this finding was controversial because up-regulated expression was detected in the tumor-flanking epithelium and upon respiratory inflammation. The authors found that a switch took place during lung carcinogenesis. Finally, they hypothesized that the sequential changes in DMBT1 expression in different locations reflected a time course that might indicate a possible mechanism in epithelial cancer. In addition, we also further researched the relationships between the other 5 de-regulated genes (STMN2, SPINK6, TUSC3, LOC100128054, and C8orf22 and disease progression. As reported, STMN2 could be a novel developmentally-associated marker and STMN2 could contribute to regulating the adipocyte/osteoblast balance [48]. Also STMN2 could be a novel target of beta-catenin/TCF-mediated carcinogenesis in hepatoma cells [49]. SPINK6 could be a prognostic indicator in nasopharyngeal carcinoma patients, and SPINK6 could play a critical role in promoting metastasis of nasopharyngeal carcinoma patients [50]. Moreover, TUSC3 was reported to related to the development of different cancers, such as glioblastoma, colorectal cancer, pancreatic cancer, and so on [51–53]. No items of LOC100128054 and C8orf22 were found from pubmed.

In addition, many studies have researched the different mechanisms of the PI3K-Akt signaling pathway. The PI3K/AKT/mTOR signaling pathway is well-known to play essential roles in cell proliferation, invasion, apoptosis, and angiogenesis in lung cancer [54–56]. However, numerous experiments are required to identify the real mechanisms underlying the roles of HOXA11-AS and its corresponding differentially expressed genes in NSCLC.

## Conclusion

In summary, because HOXA11-AS may be an important factor in different biological processes of lung cancer, we performed bioinformatics analyses (GO, pathway, KEGG, and network analyses) to identify differentially expressed genes and potential pathways. In this work, we systematically analyzed HOXA11-AS-related genes and their functional categorization, pathways and networks. Original data from TCGA was used to verify the relationships between the expression levels of HOXA11-AS and the de-regulated genes and clinicopathological parameters or patient survival. Based on the results, we speculated that HOXA11-AS may play an important role in NSCLC development and progression by regulating the expression of various pathways and genes, especially DOCK8 and TGF-beta pathway. However, the exact mechanism should be verified by functional experiments.

## Abbreviations

LncRNAs: long noncoding RNAs
NSCLC: non-small cell lung cancer
GO: gene ontology
KEGG: Kyoto Encyclopedia of Genes and Genomes
MSigDB: molecular signatures database
TCGA: the cancer genome atlas
FDR: false discovery rate
BP: biological process
CC: cellular component
MF: molecular function
DAVID: database for annotation, visualization and integrated discovery
